# Advantages of Early Operations in Appendicitis—Report of Cases

**Published:** 1908-09

**Authors:** Jas. L. Campbell

**Affiliations:** Professor Surgical Anatomy Atlanta School of Medicine, Surgeon to the Wesley Memorial Hospital; Tabernacle Infirmary and the Hospital of the Atlanta School of Medicine


### ADVANTAGES OF EARLY OPERATIONS IN APPENDICITIS—REPORT OF CASES.

BY JAS. L. CAMPBELL, M. D., PROFESSOR SURGICAL ANATOMY ATLANTA SCHOOL OF MEDICINE, SURGEON TO THE WESLEY MEMORIAL HOSPITAL; TABERNACLE INFIRMARY AND THE HOSPITAL OF THE ATLANTA SCHOOL OF MEDICINE.

It was my intention to read a paper on this subject before the Medical Association of Georgia, giving a review of the literature and statistics presented by the Association, thus showing the advance made in the past fifteen years, but I was unable to attend the Fitzgerald meeting.

Various opinions have been expressed in papers presented to the Association. Medical treatment has been urged as earnestly as surgical. And, if we had no other literature to guide us, it would be difficult for us to form an opinion except that the patient should ultimately be operated on. That, however, is not the point that gives us so much trouble. The great question that vexes us most, when we are called to see an acute case, is what to advise the family when asked if we believe the patient will die if not operated on.

My opinion is that every case should be operated on in the first twenty-four hours—certainly in the first forty-eight. If, however, the case is in good condition and has passed the latter of these periods, the patient will be given a better chance if the operation is postponed until the attack subsides. The resisting powers, especially the nerve centers, are greatly disturbed by the poison and pain, and to still further lower them by the operation will lesson the chances of recovery. After forty-eight hours the case should be treated medically and an interval operation urged, or if an abscess is in process of formation give time for the pus to be well walled in. Of course, this cannot be an iron-clad rule.

1. If the consent of the patient can be secured for an early operation, they can be saved much trouble, for in the first place 75 per cent. of the cases will recur, or the first attack may not subside until a relapse occurs, or it may become chronic.

2. In 904 cases operated on in Atlanta, 107 were for abscess. When an abscess results the patient is necessarily confined to the bed for a long period of time, to say nothing of the danger and the adhesions that will be carried through life.

3. Seven per cent. of these cases mentioned, had general peritonitis, of which several died, and those that recovered will also carry many intestinal adhesions to their graves and are in constant danger of intestinal obstruction.

4. The patient, in a few virulent cases, will, in a short time, be so saturated with poison that if they are not operated on and drained at an early stage, death is sure to result.

5. The patient is in constant danger of septic emboli being carried to some or all of the viscera where abscess and pyaemia, pneumonia, nephritis, endo or peri-carditis, in fact, many other conditions may develop.

6. No man, no matter how much experience he may have had, can tell what the condition of the appendix is. If the patient is in an acute attack, "who can say that there will ever be an interval? The patient may die in this attack."

Dr. Nicholson is fond of relating a case he operated on at St. Joseph's. The patient came down to the Hospital in a hack and he only operated because the patient insisted. He found the appendix filled with pus.

In two cases recently, in my own experience, at the Wesley Memorial Hospital, I have been very much surprised. Both young men. One came into my office with a temperature of 97, pulse normal. After an examination, I advised him to be operated on. After going home, coming back to town and finally going to the hospital, where we operated, I found the infiltration extending into the colon so that we had to resect a portion of the gut. The other had "coloc," was sitting on the front porch, when I called to see another patient in the same house. I urged an operation. He got on the car and came five miles to the city, then walked to the hospital, and when we took out the appendix we found a drachm of bloody pus, an ulcer that had destroyed the mucous and muscular coats, and only the peritoneum remained; there were no adhesions and the temperature had been normal.

I fail to see why any one can oppose an early operation, when they are aware of all these and many other facts regarding the disease.

If a married woman has had one attack she should be especially urged to have an operation, as appendicitis is a grave complication of pregnancy.

To still further urge the necessity of early operations and to show how patients could be saved much time and suffering, I will report a few cases that I have operated on that were of especuliar interest to me.

*Case* 1.—E. C. Female, age 11. Had been sick four weeks with abdominal pain, especially in right side. When I saw her, she was in a bad condition. I determinted to wait, as it was a very unfavorable case. After two days, with no danger in the condition, I decided to operate and give her a chance. The abdomen was greatly distended and so tender that she could scarcely be moved. We operated on her at the house. Opening at McBurney's point, and introducing my finger, I felt a mass extending up along and behind the colon. On making slight pressure, I ruptured the abscess—fortunately near the abdominal wall, just at the upper end of the incision; the intestines were so presesd against the abdominal wall that the pus did not get into the cavity. We hastily enlarged the opening and drained the abscess. The patient was put to bed with a bad pulse, but soon reacted. After several weeks in bed, we had to make a counter drainage just below the twelfth rib. She was well in about two months, but had a large hernia where the edge of the incision had sloughed. Eighteen months later I repaired the hernia and found, much to my surprise, an appendix about 6 inches in length nearly free from adhesions and apparently normal. On making a close examination, I found one point surrounded by slight adhesions which looked like a scar. I opened it here and a small quantity of creamy pus was present. This case is interesting for two or three reasons:

1st. The patient has been well and strong for nearly eighteen months, while there was pus in an apparently healthy appendix which was giving no trouble.

2nd. Practically all the adhesions were absorbed, even those to the scar, which was quite large.

*Case 2.*—T. A., age 15. Operated on at Wesley Memorial Hospital, 1907. Had a typical attack two weeks previous. I saw him first at East Point. He had been given a full dose of morphine a short time before and was very comfortable. There was a mass in the hypogastric region and when I examined per rectum, I found that it filled up the whole pelvis, like the head of a full term baby. He had not passed his urine well and I thought the mass might be a distended bladder. I suggested that he be catheterized and as he was in good condition, wait until next day. Early the next morning the doctor called me up and said that he had spent a bad night, so we brought him in and opened the abdomen. We found an abscess, filling up the pelvis. It contained about a pint of pus. The second or third day after the operation a fecal fistula developed and the water from an enema came through the abdominal incision. After several weeks he recovered. When he had been at home about one month he had another attack of appendicitis. We brought him back to the hospital, but as he was much better, we thought we would wait and repair the hernia that he had as the result of his abscess. I operated on him as soon as he was thoroughly free from pain and soreness. Found the intestines adhered to the scar, to each other, and to the abdominal wall. After breaking up the adhesions and removing the scar, I found the appendix in the pelvis adhered by its distal end behind the rectum opposite the second or third sacral vertebra. This was evidently the starting point of the abscess. The appendix was removed and the hernia closed and sutured with linen after the Mayo method of closing umbilical hernia. He made an uneventful recovery and is now sound and well.

In another case I operated on 48 hours after the beginning of the attack the distal end of the appendix was adherent to the abdominal wall just as in this case. The point of adhesion was surrounded by lymph and was necrotic. This would evidently have resulted in an abscess.

*Case* 3.—Mr. W., age about 35. Operated on at the Wesleyan Memorial Hospital. He had been sick with the usual symptoms of appendicitis for the past three or four weeks, was very much emaciated, there was great muscular rigidity and a mass in the right iliac region. We opened the abdomen and found a small abscess filled with foul-smelling pus. While exploring the abscess cavity I pushed my finger through the necrotic wall of the carcum and a large amount of fecal matter escaped. This fistula continued for four or five weeks, although he improved very much in strength and flesh. His appetite was ravenous, and one day a convalescent patient gave him a lot of candy, which started what was apparently an acute attack of indigestion. I saw him a few hours after the attack began and found him in a profuse perspiration and greatly prostrated. One-fourth grain morphine together with strychnine and atropine relieved him, but the next day there was no discharge from the fistula and water from an enema came through clear, so we decided to open the abdomen again. There was much tenderness in the umbilical region, so we made a high incision to the left of the umbilicus. We found a general peritonitis, the intestines were tied up like snakes in a coil in the upper abdomen, also some adhesions in the pelvis. The obstruction was in the upper part of the tract. Some time after it was relieved the old fistula began to discharge again. The patient was in such bad condition that we had to close up without breaking up all the adhesions. The abdomen was filled with hot saline and about 1,000 cc. of saline given through the left median basilic vein. He reacted slowly and made an uneventful recovery and was well at the last report. I am not sure that the candy was the cause of the peritonitis and adhesions, but the attack coming on so soon after the error in diet leads up to suppose it was.

In other abscess cases that I have operated on, the patients, with one exception, have presented no especially interesting features.

In one case, I had a second abscess to develop in the upper part of the hypogastric regions and when opened it was apparently in the abdominal wall. There was no further trouble.

If the abscess is well walled off, the patient is very likely to recover, but if the infection is so virulent that nature cannot wall it off in time to limit the general infection, the patient is almost sure to die.

It looks reasonable to suppose that if these had been, and many other similar cases were, operated on early, they would have recovered without so much time wasted and so much suffering.

